# Clinical guidelines for the management of osteoporosis in China: a systematic review

**DOI:** 10.3389/fendo.2026.1943964

**Published:** 2026-10-15

**Authors:** Ting Wei, Lizheng Xu, Shangzhi Xiong, Xinyi Zhang, Maoyi Tian, Minghui Yang, Yongcheng Wang, Mingsheng Chen, Penelope Abbott, Peter K.K. Wong, Gregory S. Kolt, Lei Si

**Affiliations:** 1 School of Health Sciences, Faculty of Health, Western Sydney University, Sydney, NSW, Australia; 2 Healthcare Big Data Research Institute, School of Public Health, Cheeloo College of Medicine, Shandong University, Jinan, China; 3 The George Institute for Global Health, Faculty of Medicine and Health, University of New South Wales, Sydney, NSW, Australia; 4 Heilongjiang Provincial Key Laboratory of Trace Elements and Human Health, Harbin Medical University, Harbin, China; 5 School of Public Health, Harbin Medical University, Harbin, China; 6 Center for Endemic Disease Control, Chinese Center for Disease Control and Prevention, Harbin Medical University, Harbin, China; 7 National Health Commission Key Laboratory in Etiology and Epidemiology, Harbin Medical University, Harbin, China; 8 Department of General Practice, The Second Affiliated Hospital of Harbin Medical University, Harbin, China; 9 Department of Orthopaedics and Traumatology, Beijing Jishuitan Hospital, Beijing, China; 10 School of Health Policy and Management, Nanjing Medical University, Nanjing, China; 11 Department of General Practice, School of Medicine, Western Sydney University, Sydney, NSW, Australia; 12 Faculty of Medicine and Health, University of Sydney, Sydney, NSW, Australia; 13 Department of Rheumatology, Westmead Hospital, Sydney, NSW, Australia; 14 College of Public Health, Taipei Medical University, Taipei, Taiwan; 15 School of Health Management, Anhui Medical University, Hefei, China; 16 Translational Health Research Institute, Western Sydney University, Sydney, NSW, Australia

**Keywords:** China, guidelines, osteoporosis, osteoporotic fractures, systematic review

## Abstract

**Background:**

The rising prevalence of osteoporosis and related fractures contributes substantially to the clinical and economic burden in China, particularly in the context of an ageing population. In response, various Chinese medical specialties and organisations have developed clinical guidelines for osteoporosis management, specifically tailored to the needs of the Chinese population. This systematic review aimed to compare and evaluate the consistency and divergence among these guidelines.

**Methods:**

A systematic search of Medline, Embase, Cochrane Library, and Web of Science was conducted for English-language papers. As well, a search of Chinese databases (China National Knowledge Infrastructure and Wanfang) and Chinese government websites was conducted. The search included documents published up to 13 August 2026. Article screening and data extraction were conducted by two independent reviewers. Management guidelines for primary osteoporosis in Chinese adults were included. Exclusion criteria were guidelines for secondary osteoporosis, guidelines for osteoporotic fractures that did not address osteoporosis management, previous versions of guidelines, and management protocols specific to local hospitals, organisations, communities, or rehabilitation centres. The methodological quality of guidelines was evaluated with the Appraisal of Guidelines for Research & Evaluation II (AGREE II).

**Results:**

Our search yielded 8,100 records, from which 32 guidelines produced by over 60 professional societies across multiple medical specialties were included. While there was consensus on the definition of osteoporosis, diagnostic criteria, prevention strategies, and the inclusion of both pharmacological and non-pharmacological treatment approaches, notable differences emerged in specific treatment recommendations. These discrepancies often reflected differences in the clinical focus of individual disciplines, such as orthopaedics, endocrinology, and geriatrics, particularly regarding diagnosis, treatment or rehabilitation, and patient populations. Unlike other international guidelines, fracture liaison services and health economic considerations, including cost-effectiveness analyses, were addressed in only some of the included guidelines.

**Conclusion:**

This systematic review synthesises current osteoporosis management guidelines in China and highlights important inconsistencies in recommendations. The findings underscore the need for high-quality, context-specific research and greater incorporation of health economic evidence to inform the development of more coherent and evidence-based national guidelines.

**Systematic review registration:**

https://osf.io/j83s4/, identifier j83s4.

## Background

Osteoporosis is a common bone disorder in middle-aged and older adults characterised by increased bone fragility resulting from low bone mass and microarchitectural deterioration. It impacts individuals of all races and ethnic backgrounds, with approximately one in three women and one in five men aged 50 years and over affected (1, 2). A 2022 meta-analysis estimated the global prevalence of osteoporosis to be 19·7%, with a significantly higher prevalence in low- and middle-income countries (LMICs) than in high-income countries (HICs) (3). Population ageing is expected to substantially increase the burden of osteoporosis globally, particularly in LMICs (4, 5). A recent systematic review estimated that the prevalence of osteoporosis among Chinese adults aged 20 to 89 years in 2020 was 13·5% based on WHO diagnostic criteria (6).

The public health impact of osteoporosis is significant due to its strong association with fractures. Given the lack of symptoms, osteoporosis is often under-diagnosed and under-treated until complicated by a fracture (7). Approximately 50% of women and 20% of men aged over 50 years are expected to sustain osteoporosis-related fractures, with up to 37 million osteoporosis-related fractures occurring annually worldwide in individuals aged over 55 years (3, 8). By 2050, 50% of all hip fractures worldwide are expected to occur in the Asian population, with China projected to be one of the most affected countries due to its large and growing elderly population (9). Osteoporotic fractures are a major cause of disability, mortality, reduced quality of life, and increased healthcare costs in older adults (10, 11). By 2050, the costs of osteoporosis-related fractures in China are projected to reach USD25·43 billion, and are expected to continue increasing with population ageing (11).

To address these challenges, both the Royal College of Physicians in the United Kingdom and the Bone Health and Osteoporosis Foundation (BHOF) – formerly the National Osteoporosis Foundation (NOF) in the United States published guidelines for osteoporosis prevention and treatment in 1999 (12, 13). In China, clinical practice guidelines are predominantly developed and issued by professional societies and their specialty branches, and commonly published in peer-reviewed Chinese medical journals. The first osteoporosis guideline in mainland China was developed by the Chinese Society of Osteoporosis and Bone Mineral Research in 2006, however, since then, multiple guidelines have been issued by various medical specialty societies across the country (14). These guidelines were developed from different specialty perspectives, reflecting the decentralised nature of osteoporosis guideline development in China and the ongoing evolution of the primary care system. As a result, no single guideline is universally recognised as the optimal standard for clinical practice across specialties and care settings. A systematic review comparing these guidelines may help identify inconsistencies and inform future consensus development across specialties and primary care. This review aims to compare and evaluate recommendations for the diagnosis, treatment, and monitoring of osteoporosis across Chinese clinical management guidelines to inform future consensus development.

## Methods

This systematic review was reported in accordance with the Preferred Reporting Items for Systematic Reviews (PRISMA) 2020 statement (15). The study protocol for this review was registered with Open Science Framework Registry (doi: osf.io/j83s4).

### Eligibility criteria

Eligible guidelines were clinical practice guidelines developed for Chinese adults with primary osteoporosis or at high risk of developing primary osteoporosis and addressing the management (diagnosis, treatment, or both) of primary osteoporosis. Exclusion criteria were as follows: guidelines focusing on people with secondary osteoporosis; guidelines on osteoporotic fractures that did not mention or involve osteoporosis management; previous versions of guidelines if updated versions were available; and management protocols specific to local hospitals, organisations, communities, or rehabilitation centres.

### Data sources and search strategy

A systematic search of Medline, Embase, the Cochrane Library, and Web of Science was conducted for Chinese guidelines published in English. A search of Chinese-language databases including the China National Knowledge Infrastructure (CNKI) and Wanfang was conducted, along with a grey literature search of the official websites of Chinese medical academic societies. In addition, government websites were searched for relevant clinical guidelines, including the National Health Commission of the People’s Republic of China, the Chinese Center for Disease Control and Prevention (CCDC), and the National Healthcare Security Administration. The search included documents published from inception to 13 August 2026.

Our search strategy was based on three key concepts: osteoporosis, clinical guidelines, and China. The first two concepts were applied in both English and Chinese database searches, while the concept of China was specifically included in the English-language database search. The detailed search strategy is provided in **Supplementary Material**.

### Selection process

Articles were imported into Covidence software, and bibliographic duplicates identified by software were removed prior to screening (16). Titles and abstracts were initially screened by two independent reviewers (TW and LX), followed by full-text screening by the same reviewers. Disagreements were resolved through discussion with a third reviewer (LS). Reference lists of included guidelines were also manually checked to locate guidelines that met the inclusion criteria but were not captured in the database search. A PRISMA flowchart (**Figure 1**) illustrates the selection process and reasons for exclusion at full-text review.

**Figure 1:**
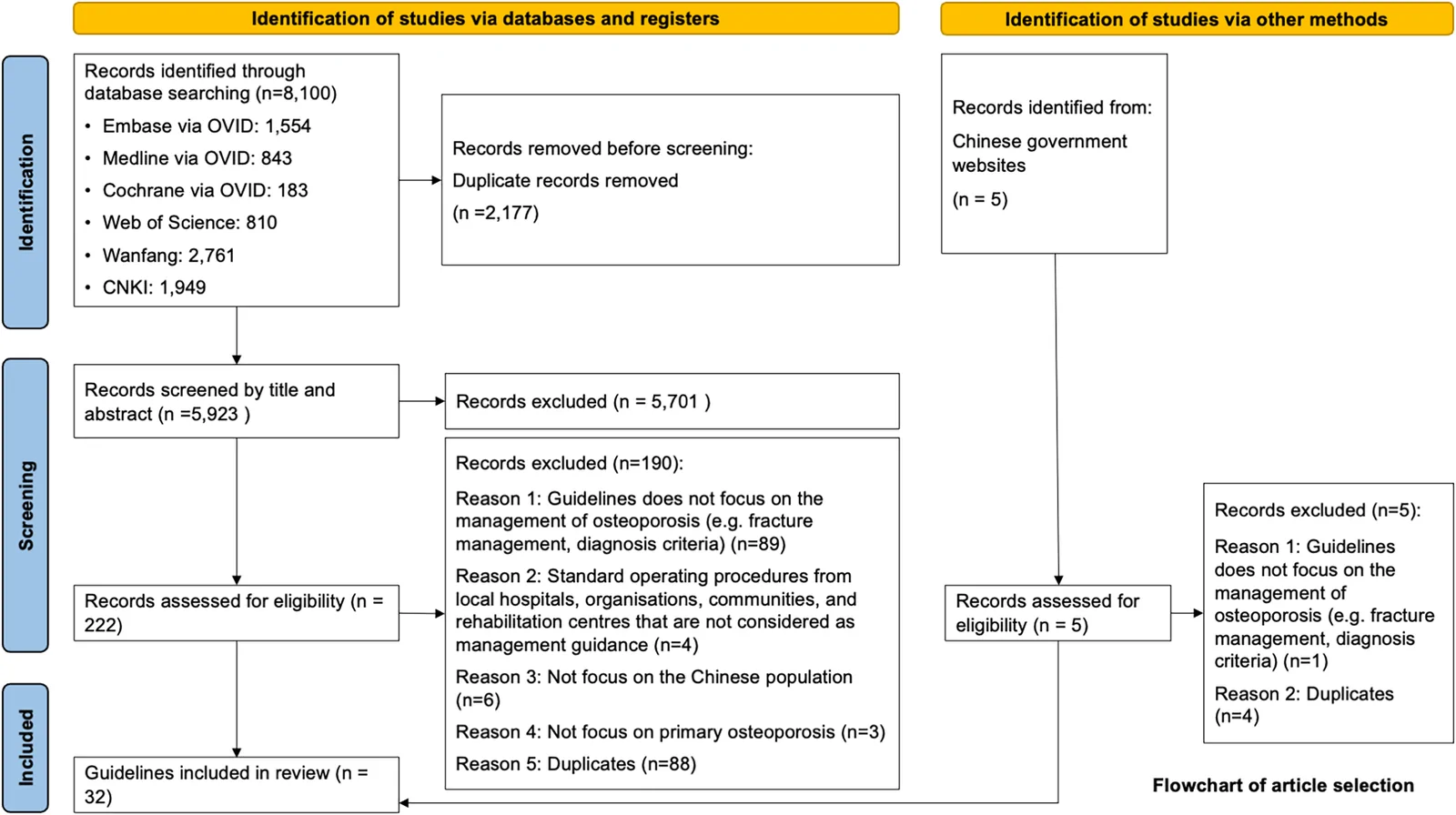
Flowchart of article selection.

### Data collection and synthesis

Data were extracted independently by two reviewers (TW and LX). The extraction form comprised eight domains: citation information, study characteristics, risk assessment for osteoporosis/osteoporotic fractures, prevention, diagnosis, treatment (both pharmacological and non-pharmacological), monitoring of osteoporosis, and other key information. The recommendations in each of the guidelines were examined to determine whether the level of evidence and strength of recommendations were reported. A full list of information included in each domain is provided in **Supplementary Material**.

We employed both thematic and content analysis approaches to analyse and summarise qualitative data. Thematic analysis was used to identify data patterns, explore themes, and examine their relationships. Findings were visualised in the osteoporosis management flow chart (**Figure 2**). Content analysis was applied to identify different treatments.

**Figure 2:**
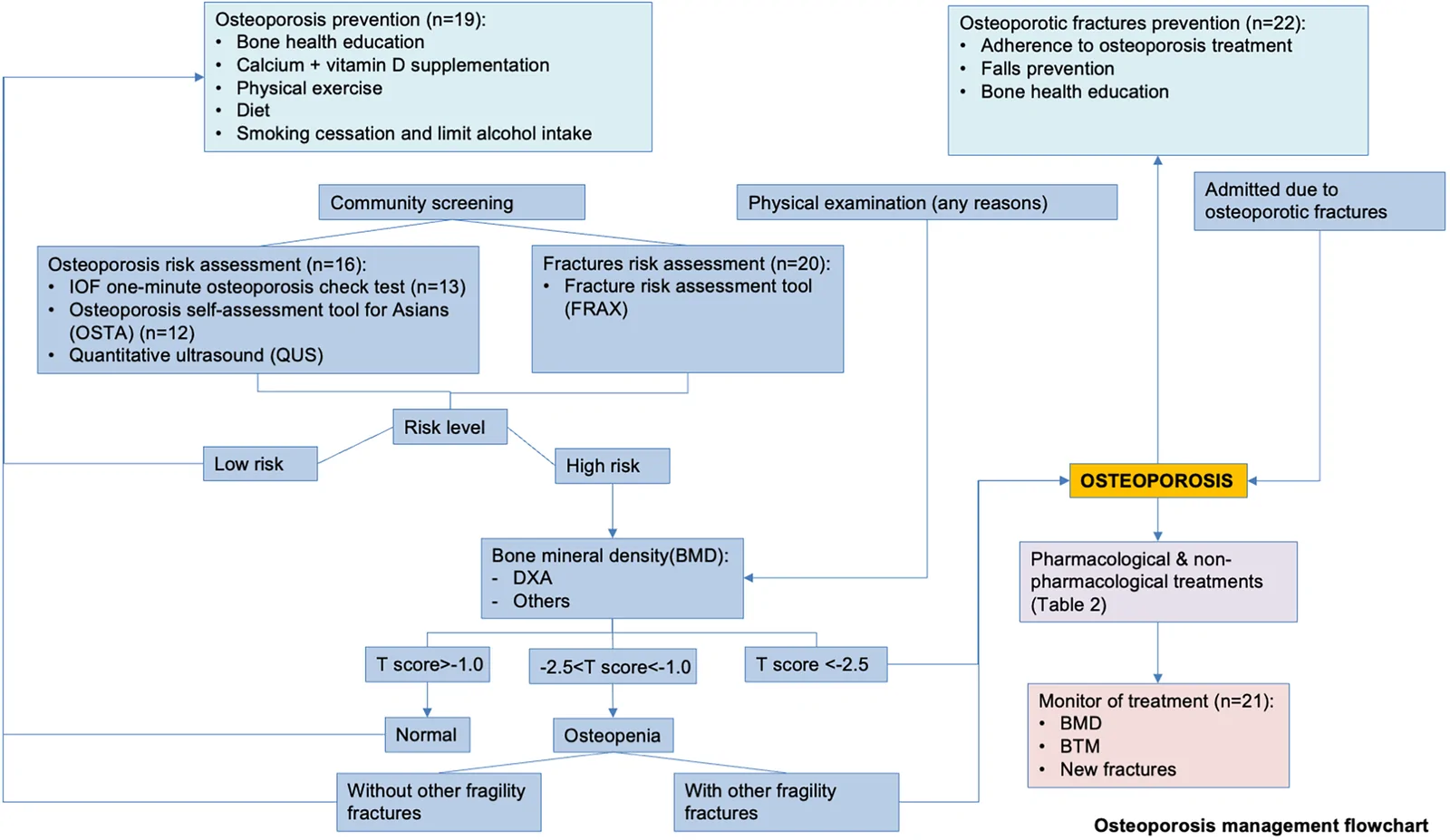
Osteoporosis management flowchart.

### Quality assessment

Methodological quality of guidelines was appraised using the Appraisal of Guidelines for Research & Evaluation II (AGREE II) tool (17), which comprises 23 items across six domains: scope and purpose, stakeholder involvement, rigour of development, clarity of presentation, applicability, and editorial independence. Two reviewers (TW and LX) independently evaluated the main text of included guidelines, excluding any supporting documents published separately, and scored each question on a scale from 1 to 7. The quality score for each domain was calculated using the standardised method suggested for the AGREE II tool (17). The mean scores for each domain were then compared between the two reviewers, and any discrepancies discussed and resolved to ensure consistency in interpretation.

## Results

A total of 8,100 records were identified from Chinese and English databases (**Figure 1**). After removing duplicates, the titles and abstracts of 5,923 articles were screened, of which 5,701 articles did not meet the inclusion criteria. Full-text screening was conducted for the remaining 222 articles, resulting in 32 guidelines being included in the final analysis.

**Table 1** presents the characteristics of the included guidelines. Except for one guideline published by Au et al. (1998) (18), all others were published after 2010, with over half (18/32) published between 2022 and 2026. Of the 32 included guidelines, 29 (91%) were developed in mainland China, two (6%) in Hong Kong (18, 19), and one (3%) in Taiwan (20). Three guidelines (18, 19, 21) were published in English, with all others (n=29, 91%) published in Chinese. The Chinese Medical Association was involved in the development of 12 (38%) guidelines (22–33), of which its Chinese Orthopaedic Association branch contributed to five (42%), and its Chinese Society of Osteoporosis and Bone Mineral Research branch contributed to three (25%). Over 60 associations from various medical specialties - such as gerontology and geriatrics, endocrinology, spine and joint health, traumatology, traditional Chinese medicine, rehabilitation medicine, general medicine, diagnostic radiology, clinical medicine - participated in guideline development. These guidelines targeted different populations, including general osteoporosis (n=11, 34%), primary osteoporosis (n=7, 22%), postmenopausal osteoporosis (n=5, 16%), osteoporosis in men (n=1, 3%), osteoporosis in the elderly (n=2, 6%), and osteoporotic fractures (n=7, 22%). The guideline focused on management of osteoporosis in men was developed by the Chinese Medical Association (24). This association also developed guidelines focused on management of general osteoporosis (26, 30, 32, 33), primary osteoporosis (22, 23, 28, 31), and osteoporotic fractures (25, 27, 29, 33). Nineteen (59%) guidelines ranked both evidence levels and grades of recommendations.

**Table 1:** Characteristics of the guidelines included (N = 32).

Title	Authors	Year	Publication	Journal	Association	Target population	Evidence level (Y/N)*	Recommendation strength (Y/N)*
Clinical Management Guidelines for Osteoporosis in Hong Kong (18)	Au et al.	1998	English	Hong Kong Medical Journal	Hong Kong Geriatrics Society; Hong Kong Society of Diagnostic Radiologists; Hong Kong College of Orthopaedic Surgeons/Hong Kong Orthopaedic Association; The Obstetrical and Gynaecological Society of Hong Kong; Society for the Study of Endocrinology, Metabolism, and Reproduction; Hong Kong College of Family Physicians; Hong Kong College of Community Medicine	Osteoporosis	N	N
Clinical Practice Guideline of Traditional Chinese Medicine for Primary Osteoporosis (21)	Xie et al.	2012	English	Chinese Journal of Integrative Medicine	Institute of Basic Research in Clinical Medicine, China Academy of Chinese Medical Sciences	Primary Osteoporosis	Y	Y
Osteoporosis Prevention and Treatment Manual for the Chinese Population, 2015 Edition (Translated) (82)	N/A	2015	Chinese	N/A	Osteoporosis Committee of China Gerontological Society (OCCGS)	Osteoporosis	N	N
Guide to Diagnosis and Treatment of Osteoporotic Fracture in China—Osteoporotic Fracture Diagnosis and Treatment Principles (83)	Qiu et al.	2018	Chinese	Heilongjiang Science	Department of Orthopedics, Chinese Academy of Medical Societies and Peking Union Medical College Hospital; Department of Orthopedics, West China Hospital; Department of Orthopedics, the First Affiliated Hospital of Chongqing Medical University; Department of Orthopedics, PLA General Hospital; Department of Orthopedics, Beijing Hospital, Ministry of Public Health; Department of Orthopedics, the First Affiliated Hospital of Soochow University; Department of Orthopedics, the Second Affiliated Hospital of Harbin Medical University	Osteoporotic Fractures	N	N
Community-based Standardized Management Algorithm of Primary Osteoporosis (84)	Du et al.	2019	Chinese	Chinese General Practice	Chinese Medical Doctor Association (CMDA)’s General Practitioners Sub-association	Primary Osteoporosis	N	N
Rehabilitation Guidelines for Osteoporosis (Translated) (34)	Shi et al.	2019	Chinese	Chinese Journal of Rehabilitation Medicine	Chinese Association of Rehabilitation Medicine (CARM); Evidence-Based Medicine Center, School of Basic Medical Sciences, Lanzhou University; Rehabilitation Information Institute of the China Rehabilitation Research Center (Translated); Gansu Province Hospital Rehabilitation Center	Osteoporosis	Y	Y
Guidelines for Integrated Traditional Chinese and Western Medicine in the Diagnosis and Treatment of Osteoporosis (Translated) (47)	Tong et al.	2019	Chinese	National Medical Journal of China	Professional Committee of Orthopedics and Traumatology of the Chinese Association of Integrative Medicine (Translated)	Osteoporosis	N	N
Guiding Principles for the Diagnosis and Treatment of Primary Osteoporosis in the Community (Translated) (22)	Zhang et al.	2019	Chinese	Chinese Journal of Osteoporosis and Bone Mineral Research	Chinese Society of Osteoporosis and Bone Mineral Research, Chinese Medical Association	Primary Osteoporosis	N	N
Practice Guideline for Patients with Osteoporosis (46)	Qiu et al.	2020	Chinese	Chinese Journal of Internal Medicine	Guangdong Province Clinical Medical Research Center for Immune Diseases	Osteoporosis	Y	Y
Traditional Chinese Medicine Diagnosis and Treatment Guidelines for Postmenopausal Osteoporosis (Osteomalacia) (2019 Edition) (Translated) (51)	Shi et al.	2020	Chinese	The Journal of Traditional Chinese Orthopedics and Traumatology	China Association of Chinese Medicine (CACM)	Postmenopausal Osteoporosis	Y	Y
Guideline for Primary Care of Primary Osteoporosis (2019) (23)	Liu et al.	2020	Chinese	Chinese Journal of General Practitioners	Chinese Medical Association; Chinese Medical Journals Publishing House; Chinese Society of General Practice, Chinese Medical Association	Primary Osteoporosis	N	N
Guidelines for Standardized Anti-osteoporosis Treatment after Fragility Fractures in China (2021) (25)	Zhang et al.	2021	Chinese	Chinese Journal of Orthopaedic Trauma	Group of Trauma Orthopaedics, Chinese Orthopaedic Association, Chinese Medical Association; Group of External Fixation and Limb Reconstruction, Chinese Orthopaedic Association, Chinese Medical Association; National Clinical Research Centre for Orthopedics, Sports Medicine & Rehabilitation	Post Fragility Fractures	Y	Y
Guideline for Rational Medication of Osteoporosis in Primary Care (26)	Zhang et al.	2021	Chinese	Chinese Journal of General Practitioners	Chinese Medical Association; Chinese Society of Clinical Pharmacy, Chinese Medical Association; Chinese Medical Journals Publishing House; Chinese Society of General Practice, Chinese Medical Association	Osteoporosis	N	N
Clinical Practice Guideline for Postmenopausal Osteoporosis with Traditional Chinese Medicine (36)	Xie et al.	2021	Chinese	China Journal of Chinese Materia Medica	Institute of Basic Research in Clinical Medicine, China Academy of Chinese Medical Sciences	Postmenopausal Osteoporosis	Y	Y
Guideline for Postoperative Rehabilitation Treatment following Vertebral Augmentation for Osteoporotic Vertebral Compression Fracture (version 2022) (42)	Xu et al.	2022	Chinese	Chinese Journal of Trauma	Spine Trauma Group of the Orthopedic Physician Branch of the Chinese Medical Doctor Association	Osteoporotic Vertebral Compression Fractures	Y	Y
Guidelines for the Diagnosis and Treatment of Primary Osteoporosis (2022) (Translated) (28)	Zhang et al.	2022	Chinese	Chinese Journal of Osteoporosis and Bone Mineral Research	Chinese Society of Osteoporosis and Bone Mineral Research, Chinese Medical Association	Primary Osteoporosis	N	N
Guidelines for the Diagnosis and Treatment of Osteoporotic Fractures (2022 Edition) (27)	Zhang et al.	2022	Chinese	Chinese Journal of Orthopaedics	Chinese Orthopaedic Association, Chinese Medical Association	Osteoporotic Fractures	N	N
Traditional Chinese Medicine Guidelines for the Diagnosis and Treatment of Osteoporotic Fractures (Translated) (85)	Shi et al.	2023	Chinese	The Journal of Traditional Chinese Orthopedics and Traumatology	China Association of Chinese Medicine (CACM)	Primary Osteoporosis	Y	Y
China Guideline for Diagnosis and Treatment of Senile Osteoporosis (2023) (37)	Wang et al.	2023	Chinese	Chinese Journal of Bone and Joint Surgery	Osteoporosis Society of China Association of Gerontology and Geriatrics; Osteoporosis Society of China International Exchange and Promotive Association for Medical and Health Care; Osteoporosis Prevention and Rehabilitation Committee of Chinese Association of Rehabilitation Medicine	Osteoporosis in the elderly	Y	Y
Clinical Intervention Guideline for Using of Anti-osteoporosis Drugs in Postoperative Fragile Hip Fractures in China (2023 Edition) (29)	Qian et al.	2023	Chinese	Chinese Journal of Joint Surgery	The Joint Surgery Branch of the Chinese Orthopaedic Association, Chinese Medical Association; Guangdong’s Medical Association of Osteoporosis and Bone Mineral Research; Foshan Shunde 3rd People’s Hospital	Fragile Hip Fractures	Y	Y
2024 OSHK Guideline for Clinical Management of Postmenopausal Osteoporosis in Hong Kong (19)	Ip et al.	2024	English	Hong Kong Medical Journal	Osteoporosis Society of Hong Kong	Postmenopausal Osteoporosis	N	N
Public Guidelines for Osteoporosis Risk Management in the Chinese Population (2024) (30)	Luo et al.	2024	Chinese	Chinese Journal of Orthopaedic Trauma	Osteoporosis Group, Chinese Association of Orthopaedic Surgeons; Youth Osteoporosis Group, Chinese Orthopaedic Association, Chinese Medical Association; Spine and Joint Health Branch, Guangdong Health Care Association; Health Communication Branch, Chinese Preventive Medicine Association; Chinese Health Risk Management Collaboration-Osteoporosis Group	Osteoporosis	Y	Y
Guideline for the Rehabilitation Treatment of Osteoporosis (2024 edition) (31)	He et al.	2024	Chinese	Chinese Journal of Evidence-based Medicine	Physical Medicine and Rehabilitation, Chinese Medical Association	Primary Osteoporosis	Y	Y
Guideline for Diagnosis and Treatment of Senile Osteoporosis with the Integrated Traditional Chinese and Western Medicine (38)	Shi et al.	2024	Chinese	Chinese Journal of Osteoporosis	The Second Affiliated Hospital of Zhejiang Chinese Medical University	Osteoporosis in the elderly	Y	Y
Guidelines for Diagnosis and Treatment of Osteoporosis in Men (24)	Zhang et al.	2025	Chinese	Chinese Journal of Osteoporosis and Bone Mineral Research; Chinese Journal of Endocrinology and Metabolism	Chinese Society of Osteoporosis and Bone Mineral Research, Chinese Medical Association	Male Osteoporosis	Y	Y
2025 Taiwan Consensus and Guidelines for the Prevention and Treatment of Osteoporosis in Adults (Translated) (20)	Huang et al.	2025	Chinese	N/A	Taiwanese Osteoporosis Association	Osteoporosis	N	N
Chinese Guideline for the Prevention and Treatment of Osteoporosis and Osteoporotic Fractures by Non⁃pharmacological Intervention (2025 Edition) (33)	Ma et al.	2025	Chinese	Chinese Journal of Orthopaedics	The Chinese Hospital Association Clinical Medical Technology Application Committee; Chinese Orthopaedic Association, Chinese Medical Association; Orthopedic Physician Branch of the Chinese Medical Doctor Association	Osteoporosis and Osteoporotic Fractures	Y	Y
Guideline for Primary Prevention of Osteoporotic Fractures (2026 edition) (39)	Tong et al.	2026	Chinese	National Medical Journal of China	Osteoporosis Prevention and Rehabilitation Committee of Chinese Association of Rehabilitation Medicine; The Osteoarthrosis Committee of Chinese Aging Well Association	Osteoporotic Fractures	Y	Y
Guidelines of Osteoporosis Screening and Health Management for Physical Examination Population (2026) (32)	Guo et al.	2026	Chinese	Chinese Journal of Health Management	Chinese Society of Health Management, Chinese Medical Association; Henan Health Management Association (Translated); Quality Management Branch of Health Service Institutions, China Health Association (CHA); Henan Key Laboratory of Chronic Disease Health Management (Translated); Henan Engineering Research Center for Clinical Proactive Health (Translated)	Osteoporosis	Y	Y
Dietary Guidelines for Adults with Osteoporosis (2026 Edition) (Translated) (35)	N/A	2026	Chinese	N/A	National Health Commission of the People’s Republic of China	Osteoporosis	N	N
Guideline for the Prevention and Management of Osteoporosis in Women with Early Menopause and Menopause (2026 Edition) (Translated) (40)	Ruan et al.	2026	Chinese	Chinese Journal for Clinicians	International Society of Gynecological Endocrinology, Chinese Branch of Gynecological Endocrinology; Fertility Protection Branch of China Health Association; Beijing Obstetrics and Gynecology Society; Osteoporosis Society of China Association of Gerontology and Geriatrics; Osteoporosis Society of China International Exchange and Promotive Association for Medical and Health Care (CPAM); Fertility Protection and Preservation Professional Committee of China Association for Promotion of Health Science and Technology; Chinese Hospital Association Obstetrics and Gynecology Hospitals Branch	Postmenopausal Osteoporosis	Y	Y
Guideline for the Integrated Traditional Chinese and Western Medicine Diagnosis and Treatment of Postmenopausal Osteoporosis (2026 Edition) (Translated) (41)	Liu et al.	2026	Chinese	Journal of Traditional Chinese Orthopedics and Traumatology	The Second Affiliated Hospital of Zhejiang Chinese Medical University	Postmenopausal Osteoporosis	Y	Y

Translation: association and titles are listed as English name/direct translate.

*Y/N represent if the recommendations have been categorized according to the level of evidence and strength of recommendation.

The recommended osteoporosis treatments included in guidelines are categorised into pharmacological and non-pharmacological treatments (**Supplementary Data Sheet 2**). Pharmacological treatments included antiresorptive, anabolic, dual action, and other therapeutic drugs. Antiresorptive medications, particularly bisphosphonates, were the most recommended pharmacological treatments and were recommended in all 26 guidelines addressing pharmacological treatment. The remaining six guidelines did not address conventional pharmacological treatment within their scope: two focused on osteoporosis rehabilitation (31, 34), and one each focused on non-pharmacological interventions (33), osteoporosis screening and health management (32), dietary management (35), and traditional Chinese medicine treatment (36). The rehabilitation guidelines provide recommendations on the types of assessments that people with osteoporosis should be subject to, including evaluations of pain levels, functional ability in daily living, quality of life, psychological function, and the risk of falls and fractures; they also cover non-pharmacological treatment options, strategies to prevent falls and fractures, and emphasise a multidimensional approach to rehabilitation including physical and psychological health, as well as patients’ awareness and understanding of osteoporosis. Parathyroid hormone analogues, calcitonin, selective estrogen receptor modulators, and menopausal hormone therapy were recommended in 23 (88%), 22 (85%), 21 (81%), and 20 (77%) of the 26 guidelines addressing pharmacological treatment, respectively. Across all 32 included guidelines, traditional Chinese medicine was recommended for osteoporosis management in 21 (66%) guidelines, including three guidelines that did not address conventional pharmacological treatment (31, 35, 36). Within the traditional Chinese medicine frameworks described in these guidelines, treatments were intended to tonify the kidney and yang, strengthen the spleen and bones, nourish the liver and kidney, and promote qi circulation and blood flow. Combination (11/32) (20, 23, 25, 27–29, 37–41) and sequential pharmacological treatment (11/32) (19, 20, 23, 25, 27–29, 37, 39, 41, 42) were also mentioned. Recommended non-pharmacological treatments included patient education (n=20, 63%), physical exercise (such as walking, jogging, stair climbing, tai chi, qigong, swimming, cycling, slow dancing, and yoga) (n=29, 91%), calcium and vitamin D supplementation (n=30, 94%), dietary modification (n=23, 72%), smoking cessation and limiting alcohol intake (n=21, 66%), and sun exposure (n=16, 50%).

**Figure 2** illustrates the stages of osteoporosis management, with four key stages identified including prevention, diagnosis, treatment, and monitoring. Based on the target population, included guidelines focused on different osteoporosis management stages. Prevention of osteoporosis was addressed in 19 guidelines, while prevention of osteoporotic fractures was discussed in 22 guidelines. The most frequently recommended osteoporosis prevention strategies included bone health education, calcium and vitamin D supplementation, physical exercise, dietary modification, and smoking cessation and limitation of alcohol intake. Adherence to osteoporosis treatment, falls prevention, and bone health education were commonly suggested as approaches to prevent osteoporotic fractures. The Fracture Liaison Service (FLS, a coordinated strategy to identify and manage patients’ post-fracture, typically involving a Fracture Liaison Coordinator and Bone Champion), was recommended in nine guidelines (18–20, 24, 28, 29, 32, 39, 42). The main diagnostic pathways for osteoporosis included community screening, physical examinations due to any reasons, and hospital admission due to osteoporotic fractures. Osteoporosis or fracture risk assessment tools, such as the International Osteoporosis Foundation (IOF) One-Minute Osteoporosis Check Test (43), the Osteoporosis Self-Assessment Tool for Asians (OSTA) (44), and the Fracture Risk Assessment Tool (FRAX) (45), as well as the quantitative ultrasound (QUS) test, were suggested as screening tools to identify high-risk populations for further investigation. The diagnosis of osteoporosis requires bone mineral density (BMD) measurement, which can also be used to monitor the effectiveness of osteoporosis treatment. Dual-energy X-ray absorptiometry (DXA) was recommended as the diagnostic tool in all guidelines that covered diagnosis of osteoporosis. Additionally, 66% of the guidelines (n=21) highlighted the importance of BMD monitoring to assess treatment response.

The AGREE-II quality appraisal results are summarised in **Table 2**. Domain 1 (Scope and Purpose) scored the highest among the six domains (mean: 87·8%; median: 91·7%; range: 63·9%-100%). This was followed by Domain 4 (Clarity of Presentation), with a mean score of 77·8%, a median score of 77·8%, and a range of 55·6%-100%. Domain 3 (Rigour of Development) had a mean score of 42·1% and a median score of 42·7% (range: 4·2%-82·3%). Fourteen (44%) guidelines described systematic methods for searching the evidence and formulating recommendations (21, 24, 25, 29–32, 34, 36–39, 41, 42). Domain 2 (Stakeholder Involvement) and Domain 6 (Editorial Independence) had mean scores of 51·7% and 42·4%, with median scores of 55·6% and 45·8%, respectively. Competing interests among guideline development group members were addressed in over half (n=18, 56%) of the guidelines (23–26, 29–34, 37–42, 46, 47), while only two (6%) provided a clear statement regarding independence from funding or sponsorship (19, 29). Although all guidelines involved clinicians in their development, only seven incorporated consumer input (31, 34, 36–38, 40, 46). The lowest scores (mean: 29·0%; median: 29·2%; range: 4·2%-56·3%) were found in Domain 5 (Applicability). Fewer than half of the guidelines provided a systematic approach for applying the recommendations.

**Table 2:** Quality assessment with domain specific scores of the guidelines using AGREE-II.

Source of guideline	Domain 1:	Domain 2:	Domain 3:	Domain 4:	Domain 5:	Domain 6:
	Scope and purpose	Stakeholder involvement	Rigour of development	Clarity of presentation	Applicability	Editorial independence
Au et al., 1998 (18)	69.4%	36.1%	4.2%	66.7%	41.7%	4.2%
Xie et al., 2012 (21)	86.1%	41.7%	38.5%	72.2%	20.8%	16.7%
N/A, 2015 (82)	91.7%	22.2%	31.3%	86.1%	29.2%	20.8%
Qiu et al., 2018 (83)	75.0%	19.4%	13.5%	63.9%	4.2%	0.0%
Du et al., 2019 (84)	100.0%	47.2%	14.6%	69.4%	18.8%	20.8%
Shi et al., 2019 (34)	100.0%	86.1%	75.0%	100.0%	56.3%	87.5%
Tong et al., 2019 (47)	63.9%	33.3%	13.5%	77.8%	20.8%	50.0%
Zhang et al., 2019 (22)	88.9%	50.0%	11.5%	55.6%	18.8%	4.2%
Qiu et al., 2020 (46)	88.9%	66.7%	41.7%	80.6%	16.7%	75.0%
Shi et al., 2020 (51)	75.0%	41.7%	53.1%	69.4%	39.6%	8.3%
Liu et al., 2020 (23)	75.0%	27.8%	10.4%	63.9%	16.7%	50.0%
Zhang et al., 2021 (25)	94.4%	63.9%	65.6%	80.6%	43.8%	75.0%
Zhang et al., 2021 (26)	72.2%	38.9%	12.5%	66.7%	16.7%	45.8%
Xie et al., 2021 (36)	97.2%	66.7%	51.0%	77.8%	10.4%	12.5%
Xu et al., 2022 (42)	91.7%	41.7%	50.0%	80.6%	8.3%	58.3%
Zhang et al., 2022 (27)	80.6%	36.1%	24.0%	80.6%	39.6%	37.5%
Zhang et al., 2022 (28)	100.0%	69.4%	75.0%	91.7%	41.7%	75.0%
Shi et al., 2023 (85)	94.4%	55.6%	16.7%	66.7%	8.3%	8.3%
Wang et al., 2023 (37)	91.7%	58.3%	69.8%	88.9%	43.8%	29.2%
Qian et al., 2023 (29)	91.7%	61.1%	62.5%	80.6%	50.0%	95.8%
Ip et al., 2024 (19)	80.6%	58.3%	29.2%	86.1%	29.2%	37.5%
Luo et al., 2024 (30)	86.1%	72.2%	69.8%	75.0%	29.2%	54.2%
He et al., 2024 (31)	94.4%	86.1%	82.3%	75.0%	39.6%	83.3%
Shi et al., 2024 (38)	91.7%	61.1%	62.5%	77.8%	45.8%	66.7%
Zhang et al., 2025 (24)	72.2%	27.8%	19.8%	66.7%	35.4%	16.7%
Huang et al., 2025 (20)	83.3%	30.6%	16.7%	66.7%	20.8%	4.2%
Ma et al., 2025 (33)	94.4%	58.3%	60.4%	86.1%	16.7%	58.3%
Tong et al., 2026 (39)	100.0%	69.4%	75.0%	86.1%	45.8%	66.7%
Guo et al., 2026 (32)	100.0%	58.3%	74.0%	88.9%	35.4%	66.7%
N/A, 2026 (35)	83.3%	30.6%	14.6%	72.2%	33.3%	16.7%
Ruan et al., 2026 (40)	100.0%	83.3%	43.8%	100.0%	31.3%	45.8%
Liu et al., 2026 (41)	94.4%	55.6%	64.6%	88.9%	18.8%	66.7%
**Median**	91.7%	55.6%	42.7%	77.8%	29.2%	45.8%
**Mean**	87.8%	51.7%	42.1%	77.8%	29.0%	42.4%

Bold text indicates the summary rows (median and mean) across all included guidelines.

## Discussion

Due to its large ageing population, China will have an increasingly significant public health burden from osteoporosis, requiring a coordinated, system-wide approach to management. This novel systematic review compared 32 published clinical guidelines for osteoporosis management in China. Despite differences in target populations and medical specialties, the included guidelines largely agreed on DXA-based diagnosis and the use of pharmacological and non-pharmacological management strategies.

Almost all included guidelines agreed on the use of DXA to measure BMD as the diagnostic tool of choice for osteoporosis. However, DXA is not widely available in China, with fewer than one DXA machine per million population (48). As a result, alternative approaches for osteoporosis assessment should be considered where access to DXA is limited. Quantitative computed tomography (QCT) may provide an alternative in selected circumstances, although its higher equipment costs and radiation exposure may limit its widespread use. These limitations may be partly addressed through opportunistic QCT, whereby CT scans obtained for other clinical indications are used for osteoporosis assessment. A nationwide multicentre study of 69,095 Chinese adults demonstrated the feasibility of this approach without additional equipment, patient time, or radiation exposure (49), while an economic evaluation found opportunistic QCT screening to be cost-effective compared with DXA (50). QUS may provide a more accessible and radiation-free option for initial osteoporosis risk assessment, particularly in resource-limited settings, although DXA remains the preferred method for definitive diagnosis.

Although the included guidelines targeted different populations, they shared many similarities in pharmacological treatment recommendations. For example, bisphosphonates were recommended in all included guidelines that addressed pharmacological treatment, although only six guidelines explicitly identified them as first-line treatment (28, 29, 38, 40, 46, 51). This differs from international guidelines, including those from the US NOF and the UK National Osteoporosis Guidelines Group (NOGG), which recommended bisphosphonates as first-line treatment (13, 52). Other pharmacological treatments, such as denosumab and romosozumab (a sclerostin inhibitor with both bone-forming and antiresorptive effects), were recommended in seventeen and eleven of the included guidelines, respectively. These frequencies should be interpreted in the context of guideline publication year, as recommendations for newer agents became more common over time: denosumab was recommended in all guidelines published from 2022 onwards, while romosozumab was first recommended in 2021 (26) and became more commonly recommended from 2023 onwards. This temporal pattern may partially reflect their more recent introduction into clinical practice; denosumab became available and reimbursed in mainland China in 2020 (53), while romosozumab was approved in mainland China in May 2026 for the treatment of osteoporosis in postmenopausal women at high risk of fracture (54). It is worth noting that bisphosphonate use is decreasing in Australia and Ireland, while use of denosumab and other newer agents is rising (55, 56). The efficacy of bisphosphonates for bone protection has been well established worldwide, including in China. Comparative evidence for teriparatide and bisphosphonates in Chinese populations is also emerging. A 2022 randomised trial in Chinese postmenopausal women found teriparatide to be non-inferior, but not superior, to alendronate for improving lumbar spine BMD (57). More recently, a 2026 multicentre randomised trial found that once weekly teriparatide resulted in significantly greater improvements in lumbar spine and total hip BMD than alendronate over 48 weeks (58). These emerging findings are consistent with the increasing role of newer osteoporosis treatments internationally, although longer-term evidence on fracture outcomes in Chinese populations remains limited. There is growing recognition of the importance of long-term treatment planning and appropriate treatment sequencing in osteoporosis management (13, 52). This is particularly relevant to denosumab, which was recommended in all included guidelines published from 2022 onwards that addressed pharmacological treatment. However, among the 17 included guidelines recommending denosumab, only three explicitly addressed its discontinuation and the need for subsequent antiresorptive treatment (19, 24, 28). Unlike bisphosphonates, for which a treatment pause may be considered in selected patients, treatment interruption or a drug holiday is not recommended with denosumab because discontinuation can result in rebound bone turnover, rapid BMD loss, and an increased risk of vertebral fractures; international guidelines therefore recommend subsequent antiresorptive treatment when denosumab is discontinued (13, 52). Future Chinese guidelines recommending denosumab should more consistently address long-term treatment planning, discontinuation risks, and appropriate sequential therapy.

Given the increasing number of pharmacological treatment options for osteoporosis, cost-effectiveness analysis is important in determining whether treatments provide sufficient benefits relative to costs. Such analyses may help inform reimbursement decisions and prioritise treatments that provide the greatest value for money. However, health economic evaluation was addressed in only two of the included guidelines (18, 19). Chinese economic evaluations have compared the cost-effectiveness of alternative pharmacological strategies, with zoledronate/zoledronic acid identified as a cost-effective option among the treatments evaluated in postmenopausal women with osteoporosis, including those at very high fracture risk (59, 60). Economic evaluations have also estimated risk-based thresholds for treatment initiation. A 2020 study estimated that zoledronate became cost-effective at a 10-year major osteoporotic fracture probability above 7%, while a recent analysis estimated drug-specific thresholds of 7% for denosumab and 12% for zoledronate (61, 62). These findings suggest that health economic evidence could inform not only the selection of pharmacological treatments but also risk-based treatment initiation thresholds in future Chinese guidelines. Further research is needed to establish robust age- and drug-specific intervention thresholds for osteoporosis treatment in China.

Use of an FLS, a multidisciplinary care model designed to prevent secondary fractures, was recommended in only 28% of the included Chinese guidelines. An FLS was associated with improved secondary fracture prevention, including lower re-fracture risk, reduced mortality, increased treatment initiation, improved medication adherence, and cost-effectiveness (63). These benefits have led to the recommendation of this model of care by both the NOF (US) and NOGG (UK) (13, 52). However, its cost-effectiveness and large-scale implementation in China require further evaluation.

Like pharmacological treatments, non-pharmacological options were recommended in all of the included guidelines. Both the US NOF and the UK NOGG guidelines strongly recommend incorporating non-pharmacological treatments, such as physical exercise, calcium and vitamin D supplementation, dietary modification, smoking cessation, and limiting alcohol intake, alongside pharmacological bone protective treatments (13, 52). Calcium and vitamin D supplementation were co-administrated alongside pharmacological interventions in most clinical trials and have generally been associated with reduced fracture risk in meta-analyses (64, 65). In addition, combining pharmacological treatment with physical exercise was also associated with greater BMD improvement than pharmacological treatment alone (66, 67). However, recommendations were not always consistent between guidelines. For example, patient education was recommended in 20 of 32 Chinese guidelines, while the NOGG guideline recommended physiotherapy-delivered education specifically for falls prevention. Previous systematic reviews of education interventions reported inconsistent findings regarding their effects on osteoporosis knowledge, medication adherence, quality of life, and physical activity levels, and all highlighted the need for high-quality RCTs (68–70). Notably, all studies included in these reviews were conducted in Western countries, which may limit the applicability of this evidence to Chinese populations.

Only one guideline (24) specifically focused on osteoporosis management in men, while the remaining guidelines addressed osteoporosis in general (primary and secondary), primary osteoporosis in both sexes, postmenopausal osteoporosis, or osteoporotic fractures. Osteoporosis is often perceived as a women’s disease; consequently, men have been less studied in clinical trials, and are more likely to be underdiagnosed and undertreated compared with women (71, 72). Lai et al. reported that 64% of currently registered osteoporosis-related randomised controlled trials included only women as participants (73). Similarly, Black et al. conducted a meta-regression analysis of 23 osteoporosis-related RCTs, of which 21 enrolled only post-menopausal women (74). Even in studies that included both women and men, women still accounted for the majority of participants; for example, a RCT conducted by Lyles et al. reported that 76% of participants were women (75, 76). Although osteoporosis or osteoporotic fractures in men are less common than in women of the same age (77, 78), the morbidity and mortality rates after osteoporotic fractures are higher in men (79, 80). Osteoporosis intervention trials in men tend to have smaller sample sizes and often use surrogate outcomes, such as BMD, rather than fractures, to assess treatment efficacy (81). This may fail to capture the true efficacy of treatments for men. More evidence-based guidelines and clinical trials tailored to osteoporosis management in men are needed.

### Strengths and limitations

This study has several strengths. First, a comprehensive bilingual search strategy was used across English and Chinese databases, supplemented by searches of relevant Chinese government websites to minimise language and publication bias. Second, to ensure the rigorous and transparent evaluation process, we adopted the AGREE II instrument to systematically assess the methodological quality of included guidelines. Finally, comparison with well-established international guidelines provided broader context for future guideline development and adaptation in China.

Despite these strengths, three specific limitations must be considered when interpreting the findings. First, the geographical distribution of the included guidelines was highly uneven, with the vast majority developed in mainland China and only three from Hong Kong or Taiwan. Formal subgroup comparisons by geographical region were therefore not undertaken. The findings primarily reflect guideline development in mainland China, while the guidelines from Hong Kong and Taiwan should be interpreted within their respective healthcare contexts, given differences in healthcare systems, regulatory frameworks, reimbursement arrangements, and treatment availability across these regions. Second, we only included guidelines that were published and accessible online, which may have excluded some internal hospital protocols or institutional documents. These sources are unlikely to significantly influence our overall conclusions. Third, there was a limitation of the evidence included in this review. The grading of evidence was identified as a notable difference between the included Chinese guidelines and the NOGG and NOF guidelines. Nineteen of the Chinese guidelines provided levels of evidence and strength of recommendation; however, most lacked a detailed description of the methods used to assess these levels. This limits comparison of recommendation strength and raises concerns about the evidence base of the recommendations.

## Conclusions

This systematic review summarises the similarities and differences among osteoporosis clinical guidelines in China. While these guidelines generally agree on the definition of osteoporosis, diagnostic criteria, and prevention strategies, there is less consensus on treatment options. Comparison with international guidelines suggests that future Chinese national guidelines should consider the widespread implementation of FLS as an effective model of care, systematic grading of evidence and the incorporation of locally generated research evidence. Furthermore, this review highlights the need for high-quality studies tailored to the Chinese context, incorporating robust economic evaluations to identify cost-effective osteoporosis management strategies. Such strategies are particularly important in the context of a large country with substantial variation in healthcare resource availability.

## Data Availability

The original contributions presented in the study are included in the article/**Supplementary Material**. Further inquiries can be directed to the corresponding author.
