# Supplementary material for: Clinical guidelines for the management of osteoporosis in China: a systematic review
Source: Front Endocrinol (Lausanne). 2026 Oct 15;17:1943964. doi: 10.3389/fendo.2026.1943964 (PMC13716593; doi:10.3389/fendo.2026.1943964)
Supplement: Supplementary file 1 [file DataSheet1.pdf]

Supplementary Material for “Clinical Guidelines for the Management of Osteoporosis in China: A Systematic Review”

Table of Contents

Appendix 1: eTable 1. PRISMA Checklist 2

Appendix 2: eTable 2. Search Strategies 6

Appendix 3: eTable 3. AGREE II Assessment Table 7

Appendix 4: Data Extraction Items 9

Appendix 1: eTable 1. PRISMA Checklist

Section and Topic	Item #	Checklist item	Location where item is reported
**TITLE**			
Title	1	Identify the report as a systematic review.	Page 1; Title page
**ABSTRACT**			
Abstract	2	See the PRISMA 2020 for Abstracts checklist.	Page 3; Abstract page
**INTRODUCTION**			
Rationale	3	Describe the rationale for the review in the context of existing knowledge.	Page 5-6; Background.
Objectives	4	Provide an explicit statement of the objective(s) or question(s) the review addresses.	Page 6; Background/3rd paragraph.
**METHODS**			
Eligibility criteria	5	Specify the inclusion and exclusion criteria for the review and how studies were grouped for the syntheses.	Page 7; Methods/ Eligibility criteria.
Information sources	6	Specify all databases, registers, websites, organisations, reference lists and other sources searched or consulted to identify studies. Specify the date when each source was last searched or consulted.	Page 7; Methods/Data sources and search strategy.
Search strategy	7	Present the full search strategies for all databases, registers and websites, including any filters and limits used.	Page 8; Methods/Data sources and search strategy. Supplementary Material Appendix 2.
Selection process	8	Specify the methods used to decide whether a study met the inclusion criteria of the review, including how many reviewers screened each record and each report retrieved, whether they worked independently, and if applicable, details of automation tools used in the process.	Page 8; Methods/Selection process.
Data collection process	9	Specify the methods used to collect data from reports, including how many reviewers collected data from each report, whether they worked independently, any processes for obtaining or confirming data from study investigators, and if applicable, details of automation tools used in the process.	Page 8; Methods/Data collection and synthesis.
Data items	10a	List and define all outcomes for which data were sought. Specify whether all results that were compatible with each outcome domain in each study were sought (e.g. for all measures, time points, analyses), and if not, the methods used to decide which results to collect.	Not applicable.
	10b	List and define all other variables for which data were sought (e.g. participant and intervention characteristics, funding sources). Describe any assumptions made about any missing or unclear information.	Page 8; Methods/Data collection and synthesis. Supplementary Material Appendix 4.

Section and Topic	Item #	Checklist item	Location where item is reported
Study risk of bias assessment	11	Specify the methods used to assess risk of bias in the included studies, including details of the tool(s) used, how many reviewers assessed each study and whether they worked independently, and if applicable, details of automation tools used in the process.	Page 9; Methods/Quality assessment.
Effect measures	12	Specify for each outcome the effect measure(s) (e.g. risk ratio, mean difference) used in the synthesis or presentation of results.	Not applicable.
Synthesis methods	13a	Describe the processes used to decide which studies were eligible for each synthesis (e.g. tabulating the study intervention characteristics and comparing against the planned groups for each synthesis (item #5)).	Page 8; Methods/Data collection and synthesis.
	13b	Describe any methods required to prepare the data for presentation or synthesis, such as handling of missing summary statistics, or data conversions.	Not applicable.
	13c	Describe any methods used to tabulate or visually display results of individual studies and syntheses.	Page 8; Methods/Data collection and synthesis.
	13d	Describe any methods used to synthesize results and provide a rationale for the choice(s). If meta-analysis was performed, describe the model(s), method(s) to identify the presence and extent of statistical heterogeneity, and software package(s) used.	Page 8; Methods/Data collection and synthesis.
	13e	Describe any methods used to explore possible causes of heterogeneity among study results (e.g. subgroup analysis, meta-regression).	Not applicable.
	13f	Describe any sensitivity analyses conducted to assess robustness of the synthesized results.	Not applicable.
Reporting bias assessment	14	Describe any methods used to assess risk of bias due to missing results in a synthesis (arising from reporting biases).	Not applicable.
Certainty assessment	15	Describe any methods used to assess certainty (or confidence) in the body of evidence for an outcome.	Not applicable.
**RESULTS**			
Study selection	16a	Describe the results of the search and selection process, from the number of records identified in the search to the number of studies included in the review, ideally using a flow diagram.	Page 10; Results/ 1st paragraph. Figure 1
	16b	Cite studies that might appear to meet the inclusion criteria, but which were excluded, and explain why they were excluded.	Page 10; Results/ 1st paragraph. Figure 1
Study characteristics	17	Cite each included study and present its characteristics.	Page 10; Results/ 2nd paragraph. Table 1
Risk of bias in studies	18	Present assessments of risk of bias for each included study.	Page 13; Results/ 5th paragraph. Table 3
Results of	19	For all outcomes, present, for each study: (a)	Table 1

Section and Topic	Item #	Checklist item	Location where item is reported
individual studies		summary statistics for each group (where appropriate) and (b) an effect estimate and its precision (e.g. confidence/credible interval), ideally using structured tables or plots.	
Results of syntheses	20a	For each synthesis, briefly summarise the characteristics and risk of bias among contributing studies.	Page 10; Results/ 2nd paragraph. Table 1
	20b	Present results of all statistical syntheses conducted. If meta-analysis was done, present for each the summary estimate and its precision (e.g. confidence/credible interval) and measures of statistical heterogeneity. If comparing groups, describe the direction of the effect.	Not applicable.
	20c	Present results of all investigations of possible causes of heterogeneity among study results.	Not applicable.
	20d	Present results of all sensitivity analyses conducted to assess the robustness of the synthesized results.	Not applicable.
Reporting biases	21	Present assessments of risk of bias due to missing results (arising from reporting biases) for each synthesis assessed.	Not applicable.
Certainty of evidence	22	Present assessments of certainty (or confidence) in the body of evidence for each outcome assessed.	Not applicable.
**DISCUSSION**			
Discussion	23a	Provide a general interpretation of the results in the context of other evidence.	Page 14; Discussion/1st paragraph.
	23b	Discuss any limitations of the evidence included in the review.	Page 19; Discussion/Strengths and Limitations.
	23c	Discuss any limitations of the review processes used.	Page 19; Discussion/Strengths and Limitations.
	23d	Discuss implications of the results for practice, policy, and future research.	Page 14-18; Discussion.
**OTHER INFORMATION**			
Registration and protocol	24a	Provide registration information for the review, including register name and registration number, or state that the review was not registered.	Page 7; Methods/1st paragraph.
	24b	Indicate where the review protocol can be accessed, or state that a protocol was not prepared.	Page 7; Methods/1st paragraph.
	24c	Describe and explain any amendments to information provided at registration or in the protocol.	Page 7; Methods/1st paragraph.
Support	25	Describe sources of financial or non-financial support for the review, and the role of the funders or sponsors in the review.	Page 21; Acknowledgements.
Competing interests	26	Declare any competing interests of review authors.	Page 21; Declaration of interests.
Availability of	27	Report which of the following are publicly available	Page 21; Data

Section and Topic	Item #	Checklist item	Location where item is reported
data, code and other materials		and where they can be found: template data collection forms; data extracted from included studies; data used for all analyses; analytic code; any other materials used in the review.	sharing statement.

Appendix 2: eTable 2. Search Strategies

**Medline via Ovid**	
**Concept 1: Osteoporosis**	
1	exp osteoporosis/ or exp osteoporosis, postmenopausal/ or exp Osteoporotic Fractures/ or exp Bone Diseases, Metabolic/
2	(osteoporosis or postmenopausal osteoporosis or (osteoporosis and postmenopausal) or osteoporotic fracture* or metabolic bone disease*).mp.
3	(low bone densit* or low bone mineral densit* or low bone mass or bone loss* or bone deterioration).mp.
4	(Osteopor* or osteopenia or osteopenic).mp.
5	(fragility fracture* or insufficiency fracture* or compression fracture*).mp.
6	1 or 2 or 3 or 4 or 5
**Concept 2: Guideline**	
7	exp Guideline/ or exp Practice Guideline/ or exp Consensus/ or exp Critical Pathways/ or exp Clinical Protocols/ or exp consensus development conference/
8	(guideline* or practice guideline* or consensus* or critical pathway* or clinical protocol* or consensus development conference*).mp.
9	exp guidelines as topic/ or exp practice guidelines as topic/ or exp Consensus Development Conferences as Topic/
10	(recommendat* or standard*).mp.
11	(position statement* or policy statement* or practice parameter* or best practice*).mp
12	((critical or clinical or practice) adj2 (path or paths or pathway or pathways or protocol*)).mp.
13	(care adj2 (standard or path or paths or pathway or pathways or map or maps or plan or plans)).mp.
14	7 or 8 or 9 or 10 or 11 or 12 or 13
**Concept 3: China**	
15	exp China/
16	(China or Chinese*).mp.
17	15 or 16
18	6 and 14 and 17

Appendix 3: eTable 3. AGREE II Assessment Table

Study	Domain 1				Domain 2				Domain 3				Domain 4				Domain 5				Domain 6			
	LX	TW	LX+TW	Scaled Domain Score	LX	TW	LX+TW	Scaled Domain Score	LX	TW	LX+TW	Scaled Domain Score	LX	TW	LX+TW	Scaled Domain Score	LX	TW	LX+TW	Scaled Domain Score	LX	TW	LX+TW	Scaled Domain Score
N/A, 2015	21	18	39	91.7%	11	3	14	22.2%	34	12	46	31.3%	21	16	37	86.1%	13	9	22	29.2%	7	2	9	20.8%
Zhang et al., 2021	19	21	40	94.4%	14	15	29	63.9%	44	35	79	65.6%	19	16	35	80.6%	16	13	29	43.8%	14	8	22	75.0%
Qiu et al., 2018	17	16	33	75.0%	10	3	13	19.4%	21	8	29	13.5%	16	13	29	63.9%	6	4	10	4.2%	2	2	4	0.0%
Du et al., 2019	21	21	42	100.0%	15	8	23	47.2%	21	9	30	14.6%	17	14	31	69.4%	8	9	17	18.8%	5	4	9	20.8%
Qiu et al., 2020	18	20	38	88.9%	17	13	30	66.7%	35	21	56	41.7%	17	18	35	80.6%	8	8	16	16.7%	14	8	22	75.0%
Shi et al., 2019	21	21	42	100.0%	16	21	37	86.1%	42	46	88	75.0%	21	21	42	100.0%	18	17	35	56.3%	14	11	25	87.5%
Xie et al., 2012	19	18	37	86.1%	10	11	21	41.7%	23	30	53	38.5%	15	17	32	72.2%	9	9	18	20.8%	4	4	8	16.7%
Zhang et al., 2021	17	15	32	72.2%	12	8	20	38.9%	17	11	28	12.5%	18	12	30	66.7%	12	4	16	16.7%	7	8	15	45.8%
Tong et al., 2019	13	16	29	63.9%	9	9	18	33.3%	18	11	29	13.5%	17	17	34	77.8%	9	9	18	20.8%	8	8	16	50.0%
Shi et al., 2023	19	21	40	94.4%	13	13	26	55.6%	13	19	32	16.7%	17	13	30	66.7%	6	6	12	8.3%	4	2	6	8.3%
Xu et al., 2022	18	21	39	91.7%	9	12	21	41.7%	28	36	64	50.0%	16	19	35	80.6%	8	4	12	8.3%	10	8	18	58.3%
Xie et al., 2021	20	21	41	97.2%	16	14	30	66.7%	29	36	65	51.0%	19	15	34	77.8%	9	4	13	10.4%	5	2	7	12.5%
Shi et al., 2020	18	15	33	75.0%	9	12	21	41.7%	33	34	67	53.1%	18	13	31	69.4%	14	13	27	39.6%	4	2	6	8.3%
Liu et al., 2020	18	15	33	75.0%	9	7	16	27.8%	13	13	26	10.4%	17	12	29	63.9%	9	7	16	16.7%	8	8	16	50.0%
Wang et al., 2023	18	21	39	91.7%	10	17	27	58.3%	42	41	83	69.8%	20	18	38	88.9%	16	13	29	43.8%	3	8	11	29.2%
Zhang et al., 2019	18	20	38	88.9%	12	12	24	50.0%	18	9	27	11.5%	17	9	26	55.6%	10	7	17	18.8%	3	2	5	4.2%
Zhang et al., 2022	19	16	35	80.6%	11	8	19	36.1%	25	14	39	24.0%	21	14	35	80.6%	16	11	27	39.6%	11	2	13	37.5%
Zhang et al., 2020	14	18	32	72.2%	9	7	16	27.8%	23	12	35	19.8%	19	11	30	66.7%	15	10	25	35.4%	4	4	8	16.7%
Zhang et al., 2022	21	21	42	100.0%	16	15	31	69.4%	43	45	88	75.0%	19	20	39	91.7%	16	12	28	41.7%	14	8	22	75.0%
Ip et al., 2024	18	17	35	80.6%	14	13	27	58.3%	29	15	44	29.2%	21	16	37	86.1%	9	13	22	29.2%	5	8	13	37.5%
Au et al., 1998	15	16	31	69.4%	5	14	19	36.1%	12	8	20	4.2%	18	12	30	66.7%	14	14	28	41.7%	3	2	5	4.2%
Huang et al., 2025	18	18	36	83.3%	8	9	17	30.6%	23	9	32	16.7%	16	14	30	66.7%	11	7	18	20.8%	3	2	5	4.2%
Luo et al., 2024	17	20	37	86.1%	18	14	32	72.2%	42	41	83	69.8%	18	15	33	75.0%	15	7	22	29.2%	9	8	17	54.2%
He et al., 2024	19	21	40	94.4%	18	19	37	86.1%	47	48	95	82.3%	16	17	33	75.0%	12	15	27	39.6%	14	10	24	83.3%
Shi et al., 2024	18	21	39	91.7%	13	15	28	61.1%	39	37	76	62.5%	17	17	34	77.8%	13	17	30	45.8%	12	8	20	66.7%
Qian et al., 2023	18	21	39	91.7%	14	14	28	61.1%	34	42	76	62.5%	17	18	35	80.6%	11	21	32	50.0%	13	14	27	95.8%
Ma et al., 2025	19	21	40	94.4%	12	15	27	58.3%	45	29	74	60.4%	20	17	37	86.1%	6	10	16	16.7%	10	8	18	58.3%
Tong et al., 2026	21	21	42	100.0%	16	15	31	69.4%	54	34	88	75.0%	21	16	37	86.1%	16	14	30	45.8%	10	10	20	66.7%
Guo et al., 2026	21	21	42	100.0%	14	13	27	58.3%	49	38	87	74.0%	21	17	38	88.9%	15	10	25	35.4%	10	10	20	66.7%
N/A, 2026	15	21	36	83.3%	8	9	17	30.6%	20	10	30	14.6%	19	13	32	72.2%	17	7	24	33.3%	4	4	8	16.7%

Ruan et al., 2026	21	21	42	100.0%	18	18	36	83.3%	35	23	58	43.8%	21	21	42	100.0%	15	8	23	31.3%	5	10	15	45.8%
Liu et al., 2026	19	21	40	94.4%	15	11	26	55.6%	44	34	78	64.6%	20	18	38	88.9%	10	7	17	18.8%	10	10	20	66.7%
Maximum possible score	42				56				112				42				56				28			
Minimum possible score	6				8				16				6				8				4			
Max-Min	36				48				96				36				48				24			

Appendix 4: Data Extraction Items

1. Basic Information
    a. Title
    b. Authors
    c. Year
    d. Journal
    e. Medical society
2. Assessment of osteoporosis risk
    a. If assessment of osteoporosis risk is mentioned in guideline
    b. If yes, which tool is suggested to assess the risk? (e.g. OSTA)
3. Assessment of fracture risk
    a. If assessment of osteoporotic fracture risk is mentioned in guideline
    b. If yes, which tool is suggested to assess the risk? (e.g. FRAX)
    c. Assessment criteria (under what condition, such assessment will be conducted)
4. Assessment frequency
    a. Age for 1st assessment
    b. Optimal interval
    c. Trigger for 1st assessment
5. Prevention method (e.g. lifestyle alteration, calcium + vd)
6. Diagnosis
    a. Tools for diagnosis
    b. Diagnosis criteria
    c. Assessment site
7. Treatment (Non-pharmacological)
    a. Patient education (y/n)
    b. Physical exercise (y/n)
    c. Ca + Vd (y/n)
    d. Diet (y/n)
    e. Other
8. Treatment (Pharmacological)
    a. Treatment trigger
    b. 1st line treatment
    c. 2nd line treatment
    d. Combined treatment
    e. Sequential treatment
9. Surgery (y/n), if yes, more information (e.g. surgery within 48 hours of hip fracture)
10. Fracture liaison services (y/n)
11. Economic evaluation (y/n)
12. Traditional Chinese medicine
    a. y/n
    b. type of medicine
13. Monitor of osteoporosis
    a. Monitor method (new fracture, BMD or BTM)
    b. Indicators for continuing, modifying and stopping treatment
    c. Medication/treatment holiday info
14. Guideline quality
    a. Recommendation level (y/n)
    b. Evidence level (y/n)
