# Supplementary material for: Clinical guidelines for the management of osteoporosis in China: a systematic review
Source: Front Endocrinol (Lausanne). 2026 Oct 15;17:1943964. doi: 10.3389/fendo.2026.1943964 (PMC13716593; doi:10.3389/fendo.2026.1943964)
Supplement: Supplementary file 2 [file DataSheet2.docx]

| **Part A. Pharmacological Management (n=26)** | | | | | | | | | | | | | | | | | | | | | | | | | | | | | | | | | | | | | | | | | | | | | | | | | | | | | | | | | | | |
| --- | --- | --- | --- | --- | --- | --- | --- | --- | --- | --- | --- | --- | --- | --- | --- | --- | --- | --- | --- | --- | --- | --- | --- | --- | --- | --- | --- | --- | --- | --- | --- | --- | --- | --- | --- | --- | --- | --- | --- | --- | --- | --- | --- | --- | --- | --- | --- | --- | --- | --- | --- | --- | --- | --- | --- | --- | --- | --- | --- |
|  | | | Au et al., 1998 | | Xie et al., 2012 | | | N/A, 2015 | | Qiu et al., 2018 | | Du et al., 2019 | | Tong et al., 2019 | | Zhang et al., 2019 | | Qiu et al., 2020 | | Shi et al., 2020 | | | Liu et al., 2020 | | Zhang et al., 2021 | | Zhang et al., 2021 | | Xu et al., 2022 | | Zhang et al., 2022 | | Zhang et al., 2022 | | Shi et al., 2023 | | | Wang et al., 2023 | | Qian et al., 2023 | | Ip et al., 2024 | | Luo et al., 2024 | | Shi et al., 2024 | | Zhang et al., 2025 | | Huang et al., 2025 | | Tong et al., 2026 | | Ruan et al., 2026 | | Liu et al., 2026 | | % | |
| **Antiresorptive drugs** | | |  | |  | | |  | |  | |  | |  | |  | |  | |  | | |  | |  | |  | |  | |  | |  | |  | | |  | |  | |  | |  | |  | |  | |  | |  | |  | |  | |  | |
| Bisphosphonates | | | ✔️ | | ✔️ | | | ✔️ | | ✔️ | | ✔️ | | ✔️ | | ✔️ | | ✔️ | | ✔️ | | | ✔️ | | ✔️ | | ✔️ | | ✔️ | | ✔️ | | ✔️ | | ✔️ | | | ✔️ | | ✔️ | | ✔️ | | ✔️ | | ✔️ | | ✔️ | | ✔️ | | ✔️ | | ✔️ | | ✔️ | | 100% | |
| RANKL inhibitors (Denosumab etc) | | |  | |  | | |  | |  | | ✔️ | |  | | ✔️ | |  | |  | | |  | | ✔️ | |  | | ✔️ | | ✔️ | | ✔️ | | ✔️ | | | ✔️ | | ✔️ | | ✔️ | | ✔️ | | ✔️ | | ✔️ | | ✔️ | | ✔️ | | ✔️ | | ✔️ | | 65% | |
| Calcitonin | | | ✔️ | | ✔️ | | | ✔️ | | ✔️ | | ✔️ | | ✔️ | | ✔️ | |  | | ✔️ | | | ✔️ | |  | | ✔️ | |  | | ✔️ | | ✔️ | | ✔️ | | | ✔️ | | ✔️ | | ✔️ | |  | | ✔️ | | ✔️ | | ✔️ | | ✔️ | | ✔️ | | ✔️ | | 85% | |
| Menopausal hormones (or Hormone replacement therapy) | | | ✔️ | | ✔️ | | | ✔️ | | ✔️ | | ✔️ | | **✔️** | | ✔️ | |  | | ✔️ | | | ✔️ | | ✔️ | |  | | ✔️ | | ✔️ | | ✔️ | | ✔️ | | |  | | ✔️ | | ✔️ | |  | |  | | ✔️ | | ✔️ | |  | | ✔️ | | ✔️ | | 77% | |
| Selective estrogen receptor modulators (SERMs) | | |  | | ✔️ | | | ✔️ | | ✔️ | | ✔️ | | ✔️ | | ✔️ | |  | |  | | |  | | ✔️ | | ✔️ | | ✔️ | | ✔️ | | ✔️ | | ✔️ | | | ✔️ | | ✔️ | | ✔️ | | ✔️ | | ✔️ | |  | | ✔️ | | ✔️ | | ✔️ | | ✔️ | | 81% | |
| **Anabolic drugs** | | |  | |  | | |  | |  | |  | |  | |  | |  | |  | | |  | |  | |  | |  | |  | |  | |  | | |  | |  | |  | |  | |  | |  | |  | |  | |  | |  | |  | |
| Parathyroid hormone analogues | | |  | | ✔️ | | | ✔️ | | ✔️ | | ✔️ | | ✔️ | | ✔️ | |  | | ✔️ | | | ✔️ | | ✔️ | | ✔️ | | ✔️ | | ✔️ | | ✔️ | | ✔️ | | | ✔️ | | ✔️ | | ✔️ | | ✔️ | | ✔️ | | ✔️ | |  | | ✔️ | | ✔️ | | ✔️ | | 88% | |
| **Dual action drugs** | | |  | |  | | |  | |  | |  | |  | |  | |  | |  | | |  | |  | |  | |  | |  | |  | |  | | |  | |  | |  | |  | |  | |  | |  | |  | |  | |  | |  | |
| Romosozumab, or Ipriflavone | | |  | |  | | |  | |  | |  | |  | |  | |  | |  | | |  | |  | | ✔️ | |  | | ✔️ | |  | | ✔️ | | | ✔️ | | ✔️ | | ✔️ | | ✔️ | | ✔️ | |  | | ✔️ | | ✔️ | |  | | ✔️ | | 42% | |
| **Other therapeutic drugs** | | |  | |  | | |  | |  | |  | |  | |  | |  | |  | | |  | |  | |  | |  | |  | |  | |  | | |  | |  | |  | |  | |  | |  | |  | |  | |  | |  | |  | |
| Vitamin D and its analogues | | | ✔️ | |  | | | ✔️ | | ✔️ | | ✔️ | | ✔️ | | ✔️ | |  | | ✔️ | | | ✔️ | |  | | ✔️ | |  | | ✔️ | | ✔️ | | ✔️ | | | ✔️ | | ✔️ | |  | |  | | ✔️ | | ✔️ | |  | | ✔️ | |  | | ✔️ | | 69% | |
| Vitamin K2 analogues | | |  | |  | | | ✔️ | | ✔️ | | ✔️ | | ✔️ | | ✔️ | |  | | ✔️ | | |  | |  | |  | |  | | ✔️ | | ✔️ | |  | | | ✔️ | | ✔️ | |  | |  | | ✔️ | | ✔️ | |  | | ✔️ | |  | |  | | 50% | |
| Strontium salts | | |  | |  | | |  | |  | | ✔️ | |  | |  | |  | | ✔️ | | |  | |  | | ✔️ | |  | |  | |  | |  | | |  | |  | |  | |  | | ✔️ | |  | |  | |  | |  | |  | | 15% | |
| Chinese Medicine | | |  | | ✔️ | | | ✔️ | | ✔️ | | ✔️ | | ✔️ | | ✔️ | |  | | ✔️ | | |  | |  | |  | | ✔️ | | ✔️ | | ✔️ | | ✔️ | | | ✔️ | | ✔️ | |  | |  | | ✔️ | | ✔️ | |  | | ✔️ | | ✔️ | | ✔️ | | 69% | |
| **Part B. Non-pharmacological Management (n=32)** | | | | | | | | | | | | | | | | | | | | | | | | | | | | | | | | | | | | | | | | | | | | | | | | | | | | | | | | | | | |
|  | Au et al., 1998 | Xie et al., 2012 | | N/A, 2015 | | Qiu et al., 2018 | Du et al., 2019 | | Shi et al., 2019 | | Tong et al., 2019 | | Zhang et al., 2019 | | Qiu et al., 2020 | | Shi et al., 2020 | | Liu et al., 2020 | | Zhang et al., 2021 | Zhang et al., 2021 | | Xie et al., 2021 | | Xu et al., 2022 | | Zhang et al., 2022 | | Zhang et al., 2022 | | Shi et al., 2023 | | Wang et al., 2023 | | Qian et al., 2023 | Ip et al., 2024 | | Luo et al., 2024 | | He et al., 2024 | | Shi et al., 2024 | | Zhang et al., 2025 | | Huang et al., 2025 | | Ma et al., 2025 | | Tong et al., 2026 | Guo et al., 2026 | N/A, 2026 | | Ruan et al., 2026 | | Liu et al., 2026 | | % |
| Patient education |  | ✔️ | |  | |  | ✔️ | | ✔️ | | ✔️ | |  | | ✔️ | | ✔️ | | ✔️ | | ✔️ |  | | ✔️ | | ✔️ | | ✔️ | | ✔️ | |  | | ✔️ | | ✔️ | ✔️ | | ✔️ | | ✔️ | |  | |  | | ✔️ | |  | | ✔️ | ✔️ |  | |  | |  | | 63% |
| Physical exercise | ✔️ | ✔️ | | ✔️ | | ✔️ | ✔️ | | ✔️ | | ✔️ | | ✔️ | | ✔️ | | ✔️ | | ✔️ | | ✔️ |  | | ✔️ | | ✔️ | | ✔️ | | ✔️ | | ✔️ | | ✔️ | |  | ✔️ | | ✔️ | |  | | ✔️ | | ✔️ | | ✔️ | | ✔️ | | ✔️ | ✔️ | ✔️ | | ✔️ | | ✔️ | | 91% |
| Ca + Vd supplement | ✔️ |  | | ✔️ | | ✔️ | ✔️ | | ✔️ | | ✔️ | | ✔️ | | ✔️ | | ✔️ | | ✔️ | | ✔️ | ✔️ | | ✔️ | | ✔️ | | ✔️ | | ✔️ | | ✔️ | | ✔️ | | ✔️ | ✔️ | | ✔️ | |  | | ✔️ | | ✔️ | | ✔️ | | ✔️ | | ✔️ | ✔️ | ✔️ | | ✔️ | | ✔️ | | 94% |
| Dietary modification |  | ✔️ | | ✔️ | |  | ✔️ | | ✔️ | | ✔️ | | ✔️ | |  | |  | | ✔️ | |  |  | | ✔️ | | ✔️ | | ✔️ | |  | | ✔️ | | ✔️ | |  | ✔️ | | ✔️ | |  | | ✔️ | | ✔️ | | ✔️ | | ✔️ | | ✔️ | ✔️ | ✔️ | | ✔️ | | ✔️ | | 72% |
| Physical therapy |  |  | | ✔️ | | ✔️ |  | | ✔️ | | ✔️ | |  | |  | | ✔️ | | ✔️ | |  |  | |  | | ✔️ | | ✔️ | | ✔️ | | ✔️ | |  | |  |  | |  | | ✔️ | |  | |  | |  | |  | |  |  |  | |  | | ✔️ | | 38% |
| Smoking cessation and limiting alcohol intake |  |  | | ✔️ | |  | ✔️ | | ✔️ | | ✔️ | | ✔️ | | ✔️ | |  | | ✔️ | | ✔️ |  | |  | | ✔️ | | ✔️ | |  | | ✔️ | | ✔️ | |  | ✔️ | | ✔️ | |  | | ✔️ | | ✔️ | | ✔️ | |  | | ✔️ |  | ✔️ | | ✔️ | | ✔️ | | 66% |
| Sun exposure |  |  | |  | |  | ✔️ | | ✔️ | | ✔️ | | ✔️ | |  | |  | | ✔️ | |  |  | |  | | ✔️ | | ✔️ | |  | | ✔️ | | ✔️ | |  | ✔️ | | ✔️ | |  | |  | |  | |  | |  | | ✔️ | ✔️ | ✔️ | | ✔️ | | ✔️ | | 50% |
| **Table 2: Pharmacological and non-pharmacological managements recommended in guidelines** | | | | | | | | | | | | | | | | | | | | | | | | | | | | | | | | | | | | | | | | | | | | | | | | | | | | | | | | | | | |
| Note: Part A includes only guidelines addressing pharmacological management within their scope. A check mark (✓) indicates that the treatment or intervention was recommended; a blank cell indicates that it was not addressed in the guideline. | | | | | | | | | | | | | | | | | | | | | | | | | | | | | | | | | | | | | | | | | | | | | | | | | | | | | | | | | | | |
